# Trust in the police and affective evaluation of police faces: a preliminary study

**DOI:** 10.3389/fpsyg.2023.1258297

**Published:** 2023-11-09

**Authors:** Nicolas M. Brunet, Natalya K. Marsh, Caitlin R. Bean, Zachary A. Powell

**Affiliations:** ^1^Department of Psychology, California State University, San Bernardino, San Bernardino, CA, United States; ^2^School of Criminology and Criminal Justice of Criminal Justice, California State University, San Bernardino, San Bernardino, CA, United States

**Keywords:** policing, valence, face perception, emotional expressions, emotional faces, affect, police legitimacy, law enforcement

## Abstract

**Introduction:**

A study was conducted to investigate if an individual’s trust in law enforcement affects their perception of the emotional facial expressions displayed by police officers.

**Methods:**

The study invited 77 participants to rate the valence of 360 face images. Images featured individuals without headgear (condition 1), or with a baseball cap (condition 2) or police hat (condition 3) digitally added to the original photograph. The images were balanced across sex, race/ethnicity (Asian, African American, Latine, and Caucasian), and facial expression (Happy, Neutral, and Angry). After rating the facial expressions, respondents completed a survey about their attitudes toward the police.

**Results:**

The results showed that, on average, valence ratings for “Angry” faces were similar across all experimental conditions. However, a closer examination revealed that faces with police hats were perceived as angrier compared to the control conditions (those with no hat and those with a baseball cap) by individuals who held negative views of the police. Conversely, participants with positive attitudes toward the police perceived faces with police hats as less angry compared to the control condition. This correlation was highly significant for angry faces (*p* < 0.01), and stronger in response to male faces compared to female faces but was not significant for neutral or happy faces.

**Discussion:**

The study emphasizes the substantial role of attitudes in shaping social perception, particularly within the context of law enforcement.

## Introduction

Trust in law enforcement is key for public cooperation in crime control and increasing police accountability (Jackson and Bradford, 2010; Tyler, 2011). Most studies of police trust use subjective assessments obtained through a survey where respondents answer questions about a general concept of police, mark their willingness to cooperate with law enforcement directives, and indicate their overall belief in police legitimacy (Goldsmith, 2005; Mazerolle et al., 2013). Survey approaches suggest one’s assessment of law enforcement impacts police-community relationships (Tyler, 2011; Mazerolle et al., 2013).

However, many people do not trust the police. Distrust of police is higher for African American and Hispanic/Latine people in the United States (Weitzer and Tuch, 2004a,b; Brunson, 2007). Media portrayals of police violence in society condition one’s views of police and result in negative assessments of law enforcement (Boudreau et al., 2019; Nägel and Nivette, 2022). Unfavorable views of police may impact one’s likelihood of complying with officer directives, participating in crime prevention efforts, or otherwise cooperating with law enforcement.

The police stand out from regular civilians because of their uniformed presence, which can be considered a priming stimulus. Priming refers to the activation of certain concepts or ideas in a person’s mind, which can influence subsequent thoughts, judgments, or behaviors (Scheufele and Tewksbury, 2007). Police attire carries strong associations and elicit thoughts and assumptions about authority, security, law enforcement and the behaviors typically associated with the police. Depending on one’s lived experience and attitude toward law enforcement, the uniform may evoke positive feelings of safety and trust, while for others it may trigger fear and anxiety.

One interesting possibility is that one’s level of trust in police can influence how they perceive the facial expressions displayed by law enforcement officers. Various factors can affect our subjective perception of faces, including early experiences (Pollak and Sinha, 2002), facial width-to-height ratio (Geniole et al., 2015) cross-race effects (Young et al., 2012), facial aging (Grondhuis et al., 2021), eyewear (Brunet and Sharp, 2020), and priming effects (Brunet, 2023). A quick glance at candidate’s faces can even predict election outcomes (Todorov et al., 2005). Additionally, personal traits and mental states can impact emotional stimuli processing. EEG studies, for example, have shown that individuals with different personality traits exhibit different neural information processing when making emotional judgments (Hamann and Canli, 2004; Fink, 2005). Anxiety levels are yet another factor that can influence emotional processing, as demonstrated by a study revealing that self-reported anxiety strongly affects attentional bias, neural activations (Fox et al., 2005), and gaze direction (Im et al., 2017), in response to threat-related stimuli. Another study (Doty et al., 2013) identified a significant positive correlation between trait anxiety, as determined by a questionnaire completed by the participants, and the ability to detect fearful faces. Based on the available research, it appears one’s social attitudes toward police may impact facial processing.

We propose that an individual’s social attitudes toward the police significantly influence how they interpret the emotional expression of law enforcement officers (Hugenberg and Bodenhausen, 2003; Hugenberg and Bodenhausen, 2004). Faces serve an important social function and pre-existing views, such as stereotypes, can inadvertently shape our perceptions of others through implicit bias. In the context of law enforcement, negative attitudes toward police may lead to harsher judgments of facial expressions. Notably, these negative judgments may be more pronounced in Black and Hispanic communities (Vogel, 2011; Kang and Bodenhausen, 2015; Peck, 2015; Bolger et al., 2021) as these populations have poor relationships with law enforcement relative to White people. Negative views of police may stem from a fear of victimization, neighborhood context, vulnerability, media-cultivated fear, or personal or vicarious experiences with police (Warr, 1990; Hagan et al., 2005; Farrall et al., 2009; Stevens and Abernethy, 2018; Miethe et al., 2019; McCarthy et al., 2020; Goffin, 2022; Pickett et al., 2022). The police may elicit feelings of anger, sadness, or depression and active prejudicial thoughts as they are considered an “outgroup” (DeSteno et al., 2004; Pichon et al., 2012). The extent of these negative attitudes can influence how one perceives emotional cues from police and non-police. The police are readily distinguishable from civilians by their distinctive uniformed appearance and the clothing, in and of itself, poses no threat to an individual. However, society ascribes meaning to clothing, recognizing it as the uniform of formal agents of social control. Consequently, an officer’s uniform may evoke latent cognitions and emotions associated with one’s preconceived notions of police. One might expect social attitudes toward police to shape one’s perception of the emotional expressions of a person enveloped in a uniform.

Our goal is to explore potential differences in how people perceive facial expressions displayed by police officers compared to those displayed by regular civilians. We accomplished this by employing manipulated face stimuli that include either a police cap or a baseball cap to simulate the appearance of police officers and regular civilians.

Charles Darwin theorized that the original egocentric functions of facial expressions adapted to serve interpersonal purposes (Knutson, 1996). Facial expressions serve important functions in social interactions and have evolved to communicate emotions. While some expressions might have originated as sensory functions, such as widening the eyes (fear) to gather visual information (Susskind et al., 2008) or narrowing the eyes to enhance target discrimination (Lee et al., 2014), they likely co-evolved to serve interpersonal functions.

In humans, the expression of fear alerts individuals to potential threats (Susskind et al., 2008), while anger indicates impending danger (Wilkowski and Meier, 2010). These affective facial expressions act as powerful alarm signals, activating the amygdala and eliciting stronger responses to negative facial expressions than to scenes depicting fearful or threatening situations. Negative valence perception is also associated with increased heart rate (Jönsson and Sonnby-Borgström, 2003), which suggests that viewing scared or angry faces activates the sympathetic nervous system.

Facial expressions also play a crucial role in police-civilian encounters by providing important social cues that can help predict the other person’s behavior. One may make judgments about intent, trust, safety, or impending violence based on facial expressions, motivating the study of factors that influence facial perception in police-civilian encounters. While one study has explored how police officers’ interpretation of facial expressions can be influenced by workload and stress (Bélanger and Blanchette, 2022), and another studies the effect of smiling police (Simpson, 2021), there is an opportunity to study how attitudes toward the police conditions facial emotion perception of officers.

Incorporating an attitudinal component to the evaluation of police faces is key for advancement of law enforcement facial perception research. Police clothing represents one’s membership with law enforcement and may lead to the activation of any associated cognitions and emotions. The association between one’s social views of police and individuals wearing a uniform are likely to be interdependent and should be accounted for by police-centered facial perception studies.

Most police officers are White males in the United States which suggests predominant views of police are shaped around one sex and ethnic identity (Goodison, 2022). One can extend this research by considering whether attitudes toward police result in unique perceptions of police faces across sex and race/ethnicity. Studying this question is critical as it will identify whether attitudes toward police are global or attenuated by those who represent law enforcement.

We utilized a photo database (Conley et al., 2018) containing images of actors expressing various emotional expressions to investigate whether police officers’ facial expressions are perceived differently. Faces were selected to balance across gender and race/ethnicity and modified in three ways for our study: leaving them without headgear (condition 1), adding a baseball cap (condition 2), and adding a police hat (condition 3). We included the baseball cap as an additional control condition to account for the possibility that visual perception could be influenced by the presence of any type of headgear, rather than a specific type of headgear (i.e., the police hat). At the end of the session, participants completed a survey about their views of police.

Prior to conducting the study, we formulated two hypotheses: (H1) faces with police hats would be perceived more negatively compared to other headgear conditions, (H2) a correlation would exist between participants’ views of the police and how faces with police hats are perceived. Our findings support H2, but not H1 (see Discussion).

## Materials and methods

### Subjects

In this study, a total of 98 undergraduate students from a Hispanic Serving Institution initially participated. However, 19 participants were ultimately excluded from the analysis for various reasons, including technical issues (3 participants), blatant disregard for instructions (5 participants), and a failure to meet the minimum criteria (rate “happy” faces, on average, at least 0.75 units higher than “neutral,” and “neutral” faces 0.75 units higher than “angry” on a 1-to-9 scale) for recognizing emotional faces (11 participants). In addition, two individuals failed to complete the survey. After excluding these 21 participants, the remaining 77 individuals were included in all aspects of the study. Their average age was 25.11 years (SD =7.1). Among them, 62 identified as female, 12 as male, and 3 as non-binary or belonging to another gender category. In terms of ethnicity, 55 participants identified as Hispanic, 11 as multi-ethnic, three as Asian, four as African American or Black, two as Caucasian, one as American Indian, and one as Hawaiian or Pacific Islander. The sample does not reflect the demographics of the American population but does resemble the larger student body at the university. The sample size aligns with those commonly observed in other studies focusing on facial perception (Davidenko, 2007; Oosterhof and Todorov, 2008; Simpson, 2019).

All participants received class credit for a psychology course in exchange for their participation. Every student gave informed consent, and no one participated more than once. Each session lasted approximately 30–40 min.

### Stimuli and experimental design

During the study, the participants sat in front of a 19-inch Dell monitor, positioned 50 cm from their head, while 360 images presented in a random sequential order at the center of the screen (17° × 23° of visual angle). Participants evaluated the valence of each image on a scale of 1 (very negative) to 9 (very positive) using the keyboard. We used one metric to avoid a multiple comparisons problem across several outcome variables. Once a participant evaluated the image, it disappeared from the screen, and was replaced by a 1 s inter-trial interval displaying a cross. The instructions asked participants to respond as quickly and accurately as possible, and images not evaluated within 4 s disappeared from the screen. The experimental paradigm was created using Experiment Builder (SR Research).

For this study, we selected 40 models from the Racially Diverse Stimulus Face Database, known as RADIATE (Vogel, 2011). RADIATE is an open-access database that provides a wide range of naturalistic faces representing individuals from diverse ethnic and racial backgrounds, showcasing different facial expressions. Our selection included 5 Asian female faces (models 01, 05, 06, 09, and 10), 5 Asian male faces (models 01, 04, 06, 09, and 10), 5 African American female faces (models 03, 05, 06, 12, and 18), 5 African American male faces (models 02, 08, 09, 16, and 18), 5 Latine female faces (models 02, 03, 04, 05, and 07), 5 Latine male faces (models 01, 06, 07, 09, and 10), 5 Caucasian female faces (models 04, 07, 09, 10, and 11), and 5 Caucasian male faces (models 01, 03, 04, 08, and 12). We specifically chose three expressions for each model: neutral, angry, and happy, resulting in a total of 120 images.

**Figure 1:**
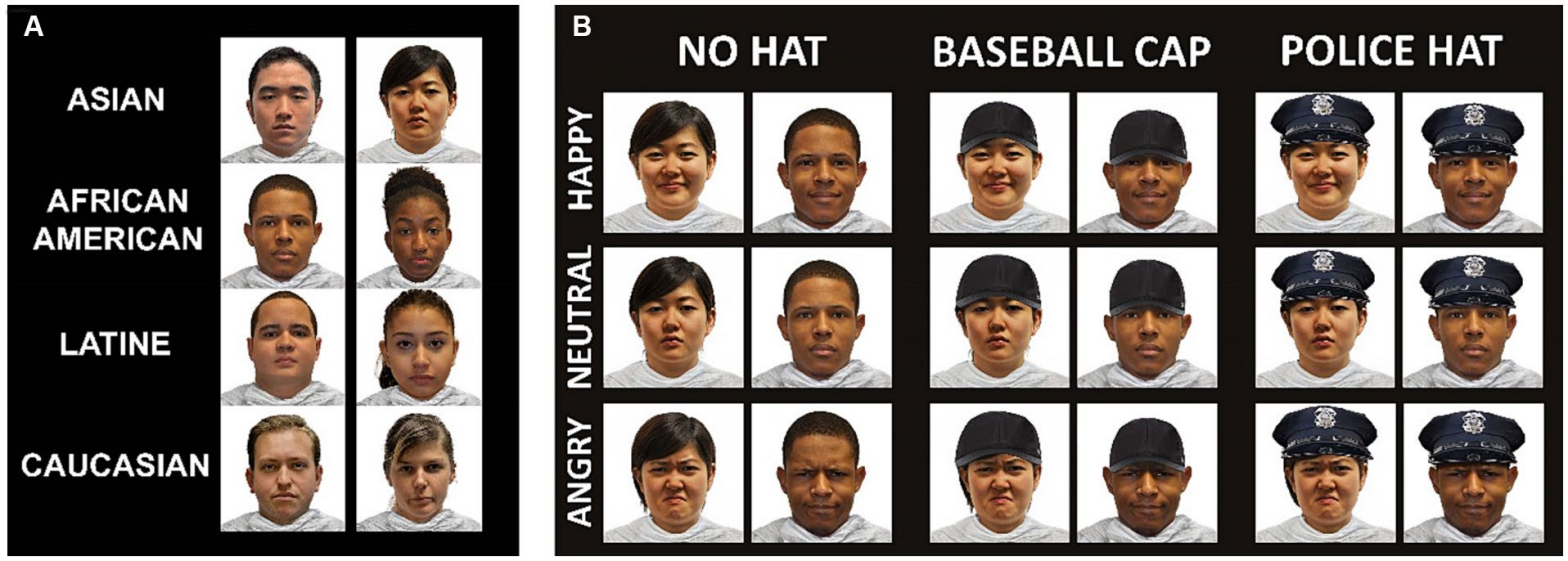
Example stimuli. Examples of the stimuli utilized in this study. Panel **(A)** presents the male and female models representing diverse racial backgrounds, including Asian, Black/African American, Hispanic/Latine, and White. Panel **(B)** showcases a female and male model with various facial expressions, including “Happy,” “Neutral,” and “Angry.” We digitally altered the original images to include baseball caps and police hats for the experimental conditions. The original images were downloaded from the open-access Racially Diverse Stimulus Face Database, RADIATE (Conley et al., 2018).

We used FaceApp to modify the hairstyle and/or facial hair of some models to ensure that the appearance of the models in our study aligned with the guidelines regarding hair length and facial hair commonly followed by police agencies. Additionally, to introduce variations to the original images (referred to as the “no hat” condition), we digitally added a baseball cap and a police hat to each image, resulting in a grand total of 360 images. Figure 1 provides examples of each condition. Following the psychophysics task, participants completed a survey aimed at exploring attitudes toward police.

Adding the control conditions is key for this study as previous facial perception research with respect to police does not account for clothing effects between images (Simpson, 2017; Simpson, 2019; Simpson, 2020; Simpson, 2021). Our experimental design also makes use of a diverse array of facial images to determine whether perceptions of police faces vary across sex and ethnicity and different facial shapes (Geniole et al., 2015). Previous studies in this area report using White male or female faces (Simpson, 2017; Simpson, 2019; Simpson, 2020; Simpson, 2021). We also compare outcomes across neutral, positive, and negative facial expressions rather than using neutral or positive expressions alone. Our careful consideration of these issues yields a more robust design that can effectively replicate and extend prior research.

### Analysis

Paired *t*-tests and pairwise correlations formed the core of the statistical analysis. The tests and statistical significance, including Cohen’s *d* effect sizes (see Table 1; Supplementary Tables S1–S5), are shown in the text, figures, table and/or figure and table captions. All analyses were performed using MATLAB. The figures were generated using MATLAB and edited in Photoshop (Adobe).

## Results

### Task performance

**Figure 2:**
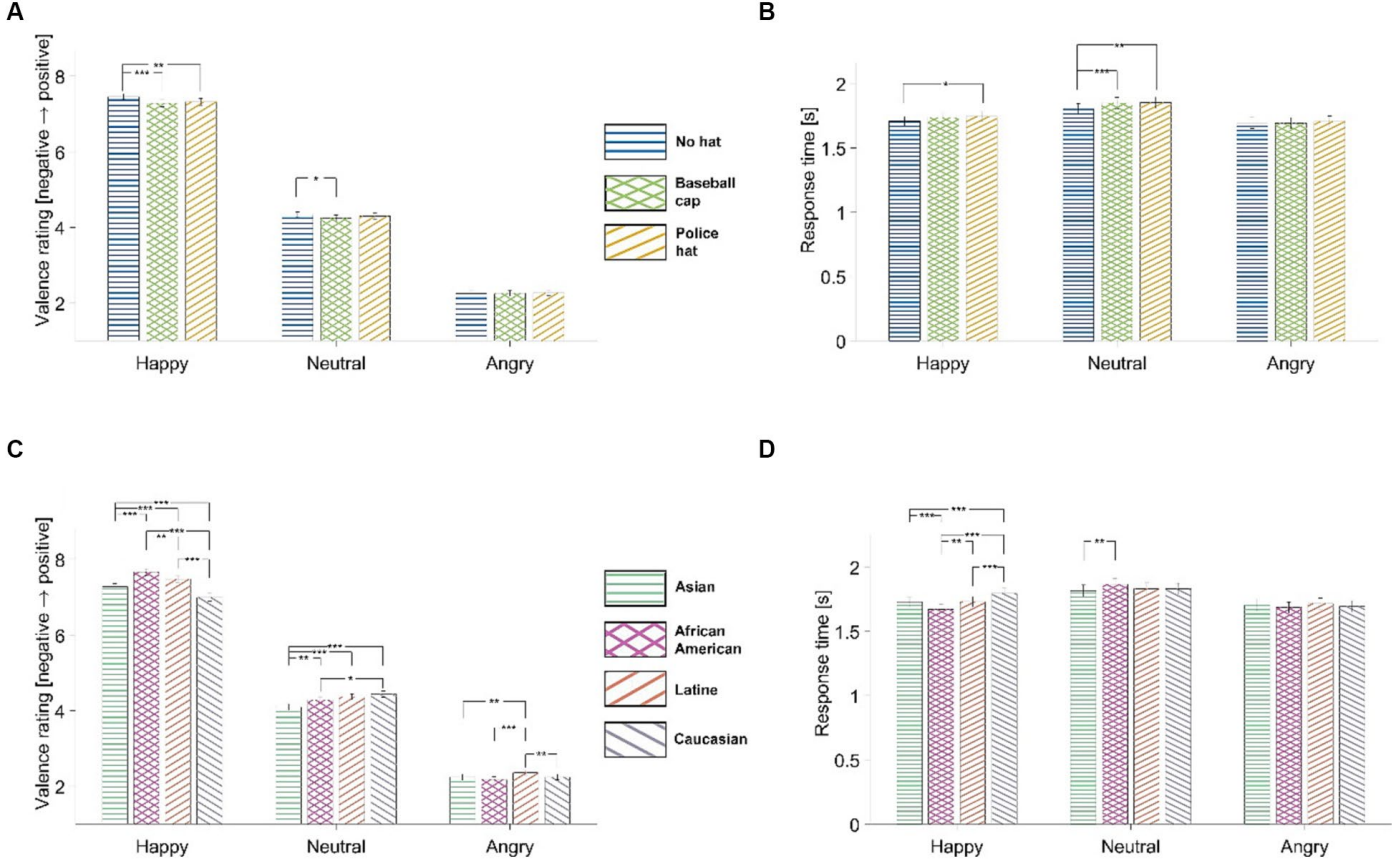
Task performance. The figure displays bar graphs illustrating valence ratings **(A)** and response time **(B)**, organized by emotional expression (“Happy,” “Neutral,” and “Angry”), averaged across all 77 participants, for each of the three experimental conditions: “No Hat” (blue horizontal hatch), “Baseball Cap” (light green crossed hatch), and “Police Hat” (light orange diagonal hatch). Panels **(C,D)** are similar to panels **(A,B)**, respectively, but incorporate the ethnicity/race variable. The facial expressions evaluated by participants were categorized as “Asian” (green horizontal hatch), “African American” (pink crossed hatch), “Latine/Hispanic” (dark orange diagonal hatch), and “White Caucasian” (gray diagonal hatch). Error bars represent standard errors. Paired-sample *t*-tests were conducted to determine statistical significance, with asterisks denoting significance levels: one, two, or three asterisks indicating *p*-values equal to or less than 0.05, 0.01, or 0.001, respectively. No significance was observed for pairs of bars without asterisks.

We used stimuli that fell into three valence categories: “happy,” “neutral,” and “angry.” To measure the participants’ task performance, we computed the average valence ratings for each category and for each experimental condition (“no hat,” “baseball cap,” and “police hat”). This gave us a total of nine values for each participant. The participants were able to distinguish between different facial expressions (see Figure 2A).

We also analyzed the average response time for each category and condition, as shown in Figure 2B. The results suggest that “angry faces” are relatively easy to identify since neither the valence assessment nor the response time were affected by the headgear type. On the other hand, happy faces were perceived as even happier when headgear was absent, indicating that hats and caps may slightly conceal the “happy” expression. Participants took longer to rate neutral faces when a hat or cap was present. Figures 2C,D display the valence ratings and response times for different racial/ethnic face categories. However, it is important to be cautious when interpreting this data, as the emotional intensity and arousal level of the models may vary slightly between different racial categories.

### Police survey

After completing the psychophysics task, participants were invited to participate in a survey aimed at assessing their views of police (Peyton et al., 2019). The survey asked respondents to map answers on a five-point Likert scale (1 = Strongly Disagree, 5 = Strongly Agree) for confidence in police, police performance, and perceived legitimacy of police using well-established measures (Sunshine and Tyler, 2003; Tyler et al., 2014; Tyler and Jackson, 2014; Peyton et al., 2019). Respondents then rated the trustworthiness (1 = Trustworthy, 7 = Untrustworthy) and compassion (1 = Compassionate, 7 = Cold-hearted) of police. Rather than test the effects of each variable, and to avoid a multiple comparison problem, we created a single score by standardizing (subtracting the mean and dividing by the standard deviation) the responses for each topic and adding them together to create a “Police Trust Score.” Standardizing each measure places items on a common scale so one can add scores from different constructs together. Our survey instrument provides a more comprehensive overview of attitudes toward police than those used in previous studies (Simpson, 2017; Peyton et al., 2019; Simpson, 2019). Interested readers should consult the Supplementary material to examine the correlations between the survey results and behavior data across each subtopic. We chose to deliver the survey after the behavioral task as several questions refer to police and might affect the behavioral task.

### Correlations between task performance and police trust survey

**Figure 3:**
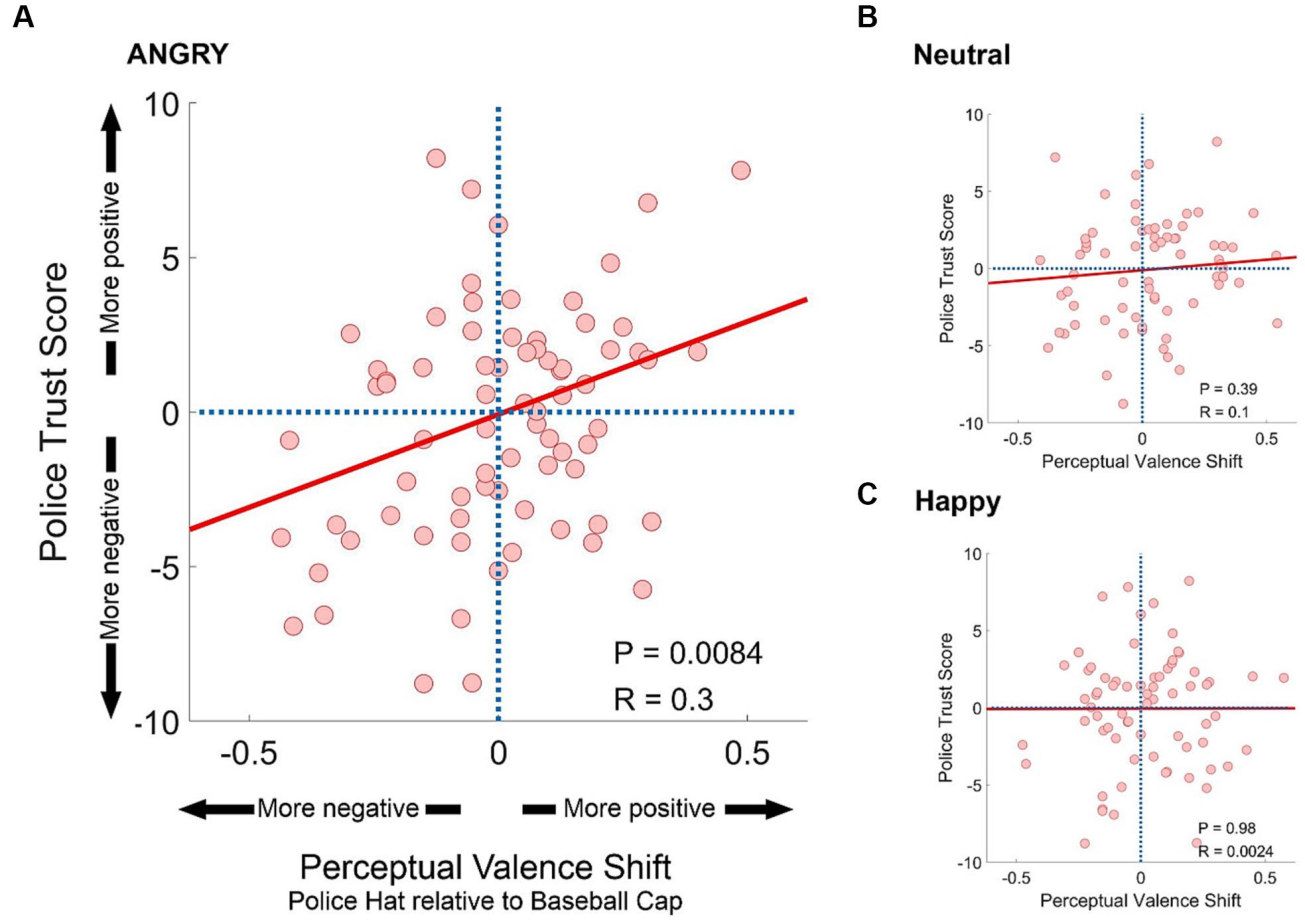
Correlation between view of police and valence ratings. Scatterplots illustrate the relationship between participants’ general views of law enforcement and their valence ratings for images featuring a police hat compared to images with a baseball cap. Each dot represents data for one participant, where the x-coordinate represents the difference in valence ratings between the experimental conditions “Police Hat” and “Baseball Cap,” and the y-coordinate represents the participant’s Police Trust Score. Positive (negative) values along the Y-axis indicate a positive (negative) view of the police, while positive (negative) values along the X-axis indicate that the participant rated images with police hats more positively (negatively) than images with baseball caps. The data were analyzed separately for responses to faces with an angry expression **(A)**, neutral expression **(B)**, and happy expression **(C)**. Least-squares lines (red lines), as well as P and R-values based upon pairwise correlations between the Police Trust Score and The Perceptual Valence Shift values (based on a sample of 77 participants) are listed for each panel. Additional findings, such as separate analysis for stimuli with male and female faces, effect sizes (Cohen’s *d*), and repeated analyses for perceptual valence shifts between each experimental condition can be viewed in Table 1. The correlation analysis results, categorized by different survey topics, are available in the Supplementary material.

The objective of our study was to investigate whether valence differences across experimental conditions correlated with police attitudes. We assessed perceptual shifts between the “Police Hat” and “Baseball Cap” conditions by calculating the difference between the average valence rating for images featuring a police hat and those featuring a baseball cap (see Figure 3). This computation resulted in 77 respondent values which we plotted against each participant’s Police Trust Score on the X and Y coordinates.

**Table 1:** Correlations between participants’ general views of law enforcement, expressed as a police trust score (see Figure 3), and differences in valence ratings among the experimental conditions “no hat,” “police hat,” and “baseball cap”.

All		Police hat–baseball cap			Police hat–no hat			Baseball cap–no hat		
	Gender	All	Male	Female	All	Male	Female	All	Male	Female
Angry	*p*-value	0.0084	0.0023	0.45	0.022	0.026	0.12	0.61	0.75	0.33
	*R*-value	0.3	0.34	0.087	0.26	0.25	0.18	0.06	−0.036	0.11
	Cohen’s *d*	0.63	0.72	0.17	0.54	0.52	0.37	0.12	0.072	0.22
Neutral	*p*-value	0.39	0.056	0.41	0.068	0.005	0.94	0.24	0.36	0.34
	*R*-value	0.1	0.22	−0.10	0.21	0.32	0.008	0.13	0.11	0.11
	Cohen’s *d*	0.2	0.45	0.19	0.43	0.68	0.016	0.26	0.22	0.22
Happy	*p*-value	0.98	0.57	0.49	0.56	0.97	0.28	0.49	0.58	0.53
	*R*-value	0.0024	−0.065	0.08	0.068	−0.0039	0.12	0.08	0.064	0.072
	Cohen’s *d*	0.0048	0.13	0.16	0.14	0.0078	0.24	0.16	0.13	0.14

The data were analyzed separately for responses to faces with an angry expression (top rows), neutral expression (middle rows), and happy expression (bottom rows). The strength of the correlation, indicated by *p* and *R*-values, derived from pairwise correlations between the Police Trust Score and Perceptual Valence Shift, are provided for each combination. The effect size (Cohen’s *d*) was computed as follow: d = 2*R/sqrt (1 − R^2). Significant correlations are highlighted in yellow (*p* < 0.05) or orange (*p* < 0.01) asterisks. The results listed in Table 1, segmented into five topics (“Performance,” “Legitimacy,” “Confidence,” “Trust,” and “Compassion”), are listed in Supplementary Tables S1–S5.

We observed significant correlations between the perceptual shift and the participants’ attitudes for “Angry” faces (see Figure 3A), but not for “Neutral” (see Figure 3B) and “Happy” faces (see Figure 3C). Table 1 provides a comprehensive summary of all *p* and *R* values, as well as Cohen’s *d* values for the correlation analysis between the Police Trust Score and potential perceptual shifts between headgear conditions. Additionally, the table features results obtained by separately analyzing responses to “female” and “male” faces. Furthermore, we explored the correlation between the survey responses, categorized into the topics “Performance,” “Legitimacy,” “Confidence,” “Trust,” and “Compassion,” and the corresponding behavioral data, which are available in Supplementary Tables S1–S5.

## Discussion

When considering all individuals, the valence rating for “angry faces” appears to be unaffected by the experimental condition (“No hat,” “Baseball Cap,” or “Police Hat”). However, notable differences between conditions were observed for “happy faces.” One plausible explanation for this finding is that happy faces are easily recognized compared to angry faces (D’Argembeau and Van der Linden, 2011), which aligns with the observations made in the RADIATE dataset (Conley et al., 2018). Enhanced recognition of happy faces allows for a higher level of perceptual granularity and may account for the influence of subtle variations, such as the presence of a hat or cap, in experimental conditions on valence ratings.

It is interesting to note that there were no significant differences in valence ratings between the “Baseball Cap” and “Police Hat” conditions for any of the emotional facial expression categories. This suggest that either the participants’ perception of the expression was not affected by these experimental conditions, or the perceptual shift toward more negative or positive perception of emotion were balanced among participants, and canceled each other out, resulting in the findings shown in Figure 2A. If the latter is true, it raises the question of whether participants’ attitudes toward law enforcement were related to their perceptual shifts. Specifically, whether they perceived faces with a “Police Hat” as more or less angry/happy than those with a “Baseball Cap” depended on their views of law enforcement. Interestingly, this was only observed for the “Angry Faces” category (Figure 3A) and not for “Neutral Faces” (Figure 3B) or “Happy Faces” (Figure 3C). Additionally, the correlation was stronger for angry male faces wearing a police hat compared to angry female faces (Table 1). Essentially, participants with negative views of law enforcement perceived an angry male face wearing a police hat as even angrier. These findings were also confirmed, although to a lesser extent, when comparing the “Police Hat” condition to the “no hat” control condition, with effects observed for both “Angry Faces” and “Neutral Faces” (Table 1). We also demonstrate these effects are not dependent of on the race/ethnicity of the image subject.

Our interpretation of police facial expressions may have a great impact in how we perceive police. A study evaluating the effects of facial expressions on perception of police officers for instance (Simpson, 2021) found that when police officers exhibit a smile, they are perceived as less aggressive and more accountable, approachable, competent, friendly, and respectful. Yet, in many police-civilian encounters, the police may not exhibit a friendly demeanor or interact with people who do not like police. Starting from a net negative view of police may inhibit de-escalation or conflict resolution techniques, increase the hostility of an encounter, or raise the risk of negative contact. These issues are crucial as perceived negativity increases the likelihood of one believing the police exhibit bias (Renauer and Covelli, 2011) during police-civilian encounters, especially for African American and Hispanic/Latine people. Further, negative views of police are stronger than positive views and curbs one’s satisfaction with law enforcement (Li et al., 2016). Starting at a deficit with respect to police hampers any kind of community policing or positive contact efforts. Unpacking the degree of negativity related to police face expression is critical for improving evidence-based community policing initiatives, encouraging civilian participation in crime prevention efforts, and gaining civilian compliance.

Our approach in this paper substantively advances prior facial perceptions of law enforcement research as it sought to replicate and extend past studies. The unique combination of behavioral and attitudinal research methods demonstrates the importance of using multiple methods for intriguing social questions. We also use multiple sets of faces balanced across emotion, sex, and ethnic identity to assess the sensitivity of uniform effects across those who may represent law enforcement. The control conditions used for the experimental conditions also allow one to determine if any observed effects are attributed to a particular type of clothing. In sum, we make several substantive contributions to literature.

Our study possesses several limitations that should be acknowledged. The participant pool consisted of college students, with a majority identifying as Hispanic/Latine, which may introduce bias into our findings. The overrepresentation of this demographic group, along with a skewed gender distribution favoring females, impacts the generalizability of our results. While these demographics have their significance, a more balanced representation would have been preferable. Furthermore, the study’s focus on Hispanic-centered research on police, while important due to its relative neglect, may limit broader applicability. A larger sample size could have allowed for a more comprehensive exploration of various factors, including age, socio-economic backgrounds, education levels, and mental health statuses, which may influence the relationships under investigation. In essence, our study serves as an important preliminary study, and we encourage additional research to address these limitations.

In future research, we will take significant steps to broaden the scope of our investigations. Given the heightened potential for volatility in interactions between police and individuals with mental health conditions such as depression, bipolar disorder, anxiety, or post-traumatic stress disorder (Kerr et al., 2010), it is crucial to expand our participant pool to include cohorts of individuals diagnosed with these mental health disorders. This expansion will enable us to delve deeper into the understanding of how mental illness impacts neural responses and physiological stress reactions triggered by the mere visual presence of law enforcement. Furthermore, we plan to adapt our research paradigm to encompass the perspectives of law enforcement officers during encounters with civilians. By considering the viewpoints of the police, we aim to study their opinions and examine their physiological changes in response to civilian stimuli. This approach will allow us to provide a comprehensive analysis of police-civilian interactions.

## Data availability statement

The raw data supporting the conclusions of this article will be made available by the authors, without undue reservation.

## Ethics statement

The studies involving humans were approved by Institutional Review Board of the California State University San Bernardino. The studies were conducted in accordance with the local legislation and institutional requirements. The participants provided their written informed consent to participate in this study. Written informed consent was obtained from the individual(s) for the publication of any potentially identifiable images or data included in this article.

## Author contributions

NB: Conceptualization, Formal analysis, Supervision, Visualization, Writing – original draft, Writing – review & editing. NM: Writing – review & editing. CB: Writing – review & editing. ZP: Conceptualization, Formal analysis, Investigation, Writing – original draft, Writing – review & editing.
