# Supplementary material for: Trust in the police and affective evaluation of police faces: a preliminary study
Source: Front Psychol. 2023 Nov 9;14:1258297. doi: 10.3389/fpsyg.2023.1258297 (PMC10666740; doi:10.3389/fpsyg.2023.1258297)
Supplement: Supplementary file 1 [file Data_Sheet_1.PDF]

Supplementary Materials

*Tables S1 to S5 below show correlations between participants' views of law enforcement (assessed using a survey and segmented quantified according to five topics: "Performance" (Table S1), "Legitimacy" (Table S2), "'Confidence" (Table S3), "Trust" (Table S4) and "Compassion" (Table S5) and the differences in valence ratings among the experimental conditions 'no hat,' 'police hat,' and 'baseball cap' (the three combinations are separated by thick borders). The data were analyzed separately for responses to faces with an angry expression (top rows), neutral expression (middle rows), and happy expression (bottom rows). The strength of the correlation, indicated by p and R-values, derived from pairwise correlations between the Police Trust Score and Perceptual Valence Shift, are provided for each combination. The effect size (Cohen's d) was computed as follow: d = 2*R/sqrt(1-R^2). Significant correlations are highlighted in yellow (p<0.05), or orange (p<0.01).*

Table S1: Performance

Performance		Police Hat - Baseball Cap			Police Hat - No Hat			Baseball Cap - No Hat		
	GENDER	All	Male	Female	All	Male	Female	All	Male	Female
Angry	P-value	0.0036	0.0051	0.21	0.027	0.045	0.11	0.89	0.65	0.53
	R-value	0.32	0.31	0.14	0.25	0.23	0.18	0.016	-0.051	0.071
	Cohen's d	0.68	0.65	0.28	0.52	0.47	0.37	0.032	0.1	0.14
Neutral	P-value	0.11	0.012	0.55	0.048	0.0026	0.94	0.56	0.82	0.48
	R-value	0.18	0.28	-0.07	0.22	0.33	0.0079	0.07	0.027	0.081
	Cohen's d	0.37	0.58	0.14	0.45	0.7	0.016	0.13	0.054	0.16
Happy	P-value	0.93	0.89	0.76	0.22	0.48	0.14	0.16	0.29	0.17
	R-value	0.0098	-0.015	0.035	0.14	0.081	0.17	0.16	0.12	0.15
	Cohen's d	0.02	0.03	0.07	0.28	0.16	0.35	0.32	0.24	0.3

Table S2: Legitimacy

Legitimacy		Police Hat - Baseball Cap			Police Hat - No Hat			Baseball Cap - No Hat		
	GENDER	All	Male	Female	All	Male	Female	All	Male	Female
Angry	P-value	0.039	0.036	0.43	0.065	0.22	0.073	0.67	0.52	0.23
	R-value	0.23	0.24	0.09	0.21	0.14	0.2	0.048	-0.073	0.14
	Cohen's d	0.47	0.49	0.18	0.43	0.28	0.41	0.096	0.15	0.28
Neutral	P-value	0.49	0.039	0.14	0.24	0.032	0.77	0.53	0.87	0.23
	R-value	0.079	0.23	-0.17	0.13	0.24	-0.034	0.07	-0.018	0.14
	Cohen's d	0.16	0.47	0.35	0.26	0.49	0.068	0.14	0.036	0.28
Happy	P-value	0.48	1	0.22	0.54	0.96	0.3	0.9	0.95	0.89
	R-value	0.081	-0.0002	0.14	0.07	0.0055	0.12	0.015	0.0073	0.015
	Cohen's d	0.16	0.00	0.28	0.14	0.011	0.24	0.03	0.015	0.03

Table S3: Confidence

Confidence		Police Hat - Baseball Cap			Police Hat - No Hat			Baseball Cap - No Hat		
	GENDER	All	Male	Female	All	Male	Female	All	Male	Female
Angry	P-value	0.01	0.0042	0.46	0.016	0.014	0.13	0.52	0.96	0.35
	R-value	0.29	0.32	0.085	0.27	0.28	0.17	0.074	-0.0063	0.11
	Cohen's d	0.61	0.68	0.17	0.56	0.58	0.35	0.15	0.013	0.22
Neutral	P-value	0.28	0.037	0.38	0.015	0.0057	0.32	0.1	0.61	0.034
	R-value	0.12	0.24	-0.10	0.27	0.31	0.11	0.19	0.058	0.24
	Cohen's d	0.24	0.49	0.20	0.56	0.65	0.22	0.39	0.12	0.49
Happy	P-value	0.48	0.57	0.59	0.2	0.45	0.13	0.35	0.73	0.23
	R-value	0.081	0.065	0.063	0.15	0.087	0.17	0.11	0.04	0.14
	Cohen's d	0.16	0.13	0.13	0.3	0.17	0.35	0.22	0.08	0.28

Table S4: Trust

Trust		Police Hat - Baseball Cap			Police Hat - No Hat			Baseball Cap - No Hat		
	GENDER	All	Male	Female	All	Male	Female	All	Male	Female
Angry	P-value	0.048	0.0037	0.92	0.092	0.12	0.22	0.76	0.31	0.18
	R-value	0.22	0.32	-0.012	0.19	0.18	0.14	0.035	-0.12	0.15
	Cohen's d	0.45	0.68	0.024	0.39	0.37	0.28	0.07	0.24	0.3
Neutral	P-value	0.7	0.19	0.33	0.12	0.0029	0.64	0.16	0.087	0.65
	R-value	0.044	0.15	-0.11	0.18	0.33	-0.053	0.16	0.19	0.052
	Cohen's d	0.088	0.3	0.22	0.37	0.7	0.11	0.32	0.39	0.1
Happy	P-value	0.82	0.29	0.39	0.85	0.93	0.67	0.68	0.29	0.81
	R-value	-0.026	-0.12	0.099	0.021	-0.0099	0.049	0.047	0.12	-0.028
	Cohen's d	0.052	0.24	0.20	0.042	0.02	0.098	0.094	0.24	0.056

Table S5: Compassion

Compassion		Police Hat - Baseball Cap			Police Hat - No Hat			Baseball Cap - No Hat		
	GENDER	All	Male	Female	All	Male	Female	All	Male	Female
Angry	P-value	0.12	0.05	0.74	0.12	0.019	0.75	0.59	0.48	0.95
	R-value	0.18	0.22	0.038	0.18	0.27	0.036	0.062	0.08	0.0067
	Cohen's d	0.37	0.45	0.076	0.37	0.56	0.072	0.12	0.16	0.013
Neutral	P-value	0.49	0.72	0.5	0.4	0.36	0.71	0.81	0.52	0.79
	R-value	0.079	0.041	0.08	0.096	0.11	0.043	0.03	0.074	-0.03
	Cohen's d	0.16	0.082	0.15	0.19	0.22	0.086	0.06	0.15	0.06
Happy	P-value	0.93	0.51	0.55	0.6	0.3	0.96	0.57	0.54	0.68
	R-value	-0.0099	-0.076	0.068	-0.061	-0.12	0.0063	-0.066	-0.071	-0.048
	Cohen's d	0.02	0.15	0.14	0.12	0.24	0.013	0.13	0.14	0.096
